# Extracellular Vesicles Derived from VEGF mRNA-Engineered Mesenchymal Stem Cells Promote Endothelial Cell Survival

**DOI:** 10.3390/cells15080717

**Published:** 2026-04-18

**Authors:** Cuiping Zhang, Peng Huang, Matthew Pak, Jennifer A. Korchak, Abba C. Zubair

**Affiliations:** Department of Laboratory Medicine and Pathology, Mayo Clinic, Jacksonville, FL 32224, USA; zhang.cuiping@mayo.edu (C.Z.); huang.peng@mayo.edu (P.H.); pak.matthew@mayo.edu (M.P.); jenniferkorchak@gmail.com (J.A.K.)

**Keywords:** extracellular vesicles, VEGF, mesenchymal stem cell, ischemic diseases, apoptosis, angiogenesis

## Abstract

**Highlights:**

**What are the main findings?**

- EVs from MSCs engineered with *VEGF* mRNA show increased VEGF mRNA and protein, while preserving standard EV size and surface markers.
- Extracellular vesicles from MSCs engineered with *VEGF* mRNA are more effective at protecting HUVECs from apoptosis than those from control MSCs.

**What are the implications of the main findings?**

- The study demonstrates that VEGF-loaded EVs can be engineered by transfecting *VEGF* mRNA into parent MSCs.
- This approach provides an innovative platform to enhance MSC-EV-based therapies for vascular-related conditions and diseases.

**Abstract:**

Extracellular vesicles derived from mesenchymal stem cells (MSC-EVs) exhibit great therapeutic potential in ischemia-associated conditions and diseases such as myocardial infarction, ischemic stroke, and wound healing. Enhancing the therapeutic efficacy of MSC-EVs could advance their clinical application. Diverse cargos (proteins, mRNA, microRNA, etc.) in MSC-EVs contribute to the therapeutic effects in various diseases. Vascular endothelial growth factor (VEGF) is one of the primary driving molecules in promoting angiogenesis and protecting endothelial cells lining blood vessels from apoptosis. In this study, we explored the feasibility of engineering parent MSCs with *VEGF* mRNA to potentiate therapeutic effects of their derived EVs. We first detected elevated levels of VEGF mRNA and protein in transfected MSCs and demonstrated the bioactivity of secreted VEGF by an angiogenesis assay. Furthermore, EVs derived from *VEGF* mRNA-engineered MSCs (VEGF-MSC-EVs) contained high levels of VEGF mRNA and protein and showed superior ability to protect human umbilical vein endothelial cells (HUVECs) from apoptosis compared to EVs derived from control MSCs (control MSC-EVs). To our knowledge, this is the first report demonstrating that VEGF-MSC-EVs boost therapeutic efficacy by promoting endothelial cell survival. Our findings offer a novel approach for cell-free therapy in ischemia-associated conditions and diseases.

## 1. Introduction

Mesenchymal stem/stromal cells (MSCs) have been extensively studied in regenerative therapies because of their immunomodulatory, pro-angiogenic, and anti-apoptotic properties. Current evidence strongly suggests that MSC-based therapies exert their effects primarily through the paracrine mechanisms rather than MSC differentiation capacity [1]. Among these paracrine factors, extracellular vesicles (EVs) have emerged as pivotal mediators of MSC function. EVs not only inherit the beneficial properties of MSCs, including immunosuppression and angiogenesis, but also show lower immunogenicity and the increased ability to cross biological barriers compared to their parent MSCs [2,3]. EVs are membrane-bound vesicles that carry bioactive molecules such as mRNA, microRNA, and proteins. EVs facilitate intercellular communication by transferring functional cargos from parent cells to recipient cells. Over the past decade, MSC-derived EV (MSC-EV) related studies have progressed rapidly, and MSC-EVs have shown promising therapeutic effects on a variety of diseases, including wound healing, cardiovascular diseases, and COVID-19 [4,5,6,7]. Additionally, MSC-EVs have been explored as natural carriers for the delivery of gene therapy, including RNA, DNA, proteins, and other biomolecules [3]. In our previous studies, we have developed engineered MSCs with human mRNA encoding interleukin 10 (IL-10), endothelial nitric oxide synthase (eNOS), and interleukin 6 (IL-6) to enhance the therapeutic efficacy of MSCs [8,9,10]. In this study, we investigated whether transfection of MSCs with vascular endothelial growth factor (VEGF) mRNA could significantly enhance the anti-apoptotic features of their secreted EVs.

Vascular endothelial growth factor (VEGF, referring specifically to VEGF-A) is a proangiogenic molecule that plays an indispensable role in tissue repair. The main role of VEGF in angiogenesis is to promote the growth and survival of vascular endothelial cells by interacting with mitogenic and anti-apoptotic signaling pathways. Due to alternative splicing, there are several isoforms of VEGF, including VEGF121, VEGF165, VEGF189, and VEGF206 [11]. Given that VEGF165 is the dominant isoform and is critical in angiogenic pathophysiological regulation, we selected the human mRNA transcript of VEGF165 to transfect human bone marrow-derived MSCs (BM-MSCs), thereby generating MSC-EVs to effectively induce angiogenesis and promote endothelial cells’ survival. VEGF derived from BM-MSCs has been shown to have beneficial effects for the treatment of ischemic conditions such as cardiac repair [12] and wound healing [13]. Multiple studies have reported the use of *VEGF* DNA-based engineering of MSCs [14,15,16], as well as *VEGF* mRNA-engineered MSCs in models of myocardial infarction and critical limb ischemia [17,18]. Furthermore, VEGF has also been reported to play a critical role in tissue repair functions mediated by MSC-EVs [19,20].

A variety of strategies have been explored to enhance the therapeutic efficacy of MSC-EVs, including direct cargo loading and hypoxic preconditioning [21]. In this study, we demonstrate for the first time that EVs derived from *VEGF* mRNA-engineered MSCs (VEGF-MSC-EVs) can effectively prevent apoptosis in human umbilical vein endothelial cells (HUVECs), laying the foundation for further investigation of EV-based therapies in ischemia-associated conditions and diseases.

## 2. Materials and Methods

### 2.1. Cell Culture

Bone marrow-derived mesenchymal stem cells (BM-MSCs) were isolated from commercial de-identified bone marrow (catalog# ABM002) purchased from AllCells (Alameda, CA, USA). All procedures were conducted in accordance with ethical standards. Donor informed consent and Institutional Review Board (IRB) approval for sample collection were confirmed by the supplier. The isolation procedures of BM-MSCs were performed as previously described [8]. MSCs were cultured and expanded in MSC growth medium composed of Minimum Essential Medium α (MEMα) (Gibco, Grand Island, NY, USA, catalog# 12561072) supplemented with 5% human platelet lysate (Sexton Biotechnologies, Indianapolis, IN, USA, catalog# PL-NH-500) and 1% GlutaMAX supplement (Gibco, catalog# 35050061) in a 37 °C, 5% CO_2_ humidified cell culture incubator. All MSCs used in this study were within passage 4. Primary human umbilical vein endothelial cells (HUVECs) (ATCC, Manassas, VA, USA, catalog# PCS-100-010) were cultured in HUVEC growth medium composed of Vascular Cell Basal Medium (ATCC, catalog# PCS-100-030) supplemented with the Endothelial Cell Growth Kit-VEGF (ATCC, catalog# PCS-100-014) and 0.1% Penicillin–Streptomycin solution (Gibco, catalog# 15140122). Subculturing procedures for HUVCs were performed following the manufacturer’s instructions.

### 2.2. mRNA Synthesis and Transfection

The coding sequence of human VEGFA mRNA (NCBI, NM_001171626.1, https://www.ncbi.nlm.nih.gov/) was selected for the synthesis. *VEGF* mRNA was produced by TriLink BioTechnologies (San Diego, CA, USA) using CleanCap technology. CleanCap *EGFP* mRNA (TriLink BioTechnologies, catalog# L-7601) was used to express an enhanced green fluorescent protein. All mRNA products underwent rigorous DNase treatment to remove residual DNA templates in accordance with the supplier’s quality control procedures (residual DNA < 5 ng/µg).

Lipofectamine MessengerMAX Reagent (Thermo Fisher Scientific, Carlsbad, CA, USA, catalog# LMRNA015) was used for the transfection of *VEGF* mRNA following the protocols previously established [10]. Briefly, MSCs were seeded into 6-well plates or T150 flasks, and mRNA transfection was performed when cells reached approximately 80% confluence. *VEGF* mRNA or *EGFP* mRNA and Lipofectamine transfection reagent were added to MSC culture at a final concentration of 1.25 µg/mL and 1.875 µL/mL, respectively. The control MSCs underwent the same procedure with no addition of mRNAs.

### 2.3. Reverse Transcription–Quantitative PCR (RT-qPCR)

Total RNA from MSCs was extracted using RNeasy Plus Mini Kit (Qiagen, Hilden, Germany, catalog# 74134,), and the reverse transcription was performed with QuantiNova Reverse Transcription Kit (Qiagen, catalog# 205411) according to the manufacturer’s instructions. Quantitative PCR was conducted to assess target mRNA levels using TaqMan Fast Advanced Master Mix (Applied Biosystems, Waltham, MA, USA, catalog# 4444963) and Gene Expression TaqMan Assays (Applied Biosystems) targeting *VEGFA* (Exon 3–4) (Hs00900055_m1) and *GAPDH* (Hs02758991_g1) as per the manufacturer’s protocols. The 2^−ΔΔCt^ method was used to calculate relative *VEGF* mRNA levels with *GAPDH* as the reference gene.

Total RNA from EVs was isolated using the Plasma/Serum RNA/DNA Purification Mini Kit (Norgen Biotek, Thorold, ON, Canada, catalog# 55200) according to the manufacturer’s instructions. Oligo (dT)-primed RT-qPCR was performed to selectively synthesize cDNA from polyadenylated mRNA. Briefly, 20 ng of *VEGF* mRNA construct, Hela cell total RNA, or total RNA isolated from EVs was reverse-transcribed with oligo (dT) primers using SuperScript IV First-Strand Synthesis System (Thermo Fisher Scientific, catalog#18091150) following the manufacturer’s protocol. Quantitative PCR (qPCR) was performed using two Gene Expression TaqMan Assays targeting *VEGFA*: *VEGF* (Exon 1–2) (Hs00173626_m1) and *VEGF* (Exon 3–4) (Hs00900055_m1). To quantify *VEGF* mRNA copy number, a standard curve was generated using a serial dilution of *VEGF* mRNA construct (TriLink BioTechnologies) with *VEGF* (Exon 3–4) primers, ranging from 1 × 10^8^ to 1 × 10^2^ copies per reaction. The standard curve (log 10 (*VEGF* mRNA copy number) vs. Ct) was generated using GraphPad Prism (Version 10.0.0). *VEGF* mRNA copy numbers in EV samples were normalized to total EV RNA content. All reactions were performed in triplicate.

### 2.4. Enzyme-Linked Immunosorbent Assay (ELISA)

Conditioned culture media from control MSCs or VEGF-MSCs (CCM-control or CCM-VEGF, respectively) were collected at different time points: (1) on Days 1, 2, 4, and 6 in the first time-course experiment, (2) at 4, 10, 20, and 30 h in the short time-course experiment, and (3) at 24 h after thawing MSCs that had been transfected with VEGF mRNA for 4 and 10 h. Centrifugation at 1500 rpm for 10 min at 4 °C was conducted to remove cell debris, and then the supernatant was aliquoted and immediately stored at −80 °C. In addition, ELISA was conducted to measure VEGF protein levels in EV samples (control MSC-EVs and VEGF-MSC-EVs). The samples were thawed on ice immediately before conducting the ELISA. To ensure sample concentrations fell within the standard curve, CCM-control samples were diluted at 1:10, CCM-VEGF at 1:100, control-MSC-EVs at 1:5, and VEGF-MSC-EVs at 1:50. This assay was conducted using a human VEGF ELISA kit (Aviva systems biology, San Diego, CA, USA, catalog# OKBB00271) according to the manufacturer’s instructions. For ELISA analysis of isolated EVs, VEGF protein levels were normalized to the total EV protein content, as determined using the Pierce BCA Protein Assay Kit (Thermo Fisher Scientific, catalog# 23227). Each sample was tested in duplicate or triplicate.

### 2.5. Angiogenesis Assay

To prepare conditioned culture media (CCM) for this assay, MSCs were first cryopreserved in CryoStor^®^ CS10 (StemCell Technologies, Vancouver, BC, Canada, catalog# 07930) after 4 h of transfection. VEGF-MSCs and control MSCs were seeded into the 6-well plates from cryopreservation and cultured for 24 h, after which CCM-VEGF and CCM-control samples were collected. The CCM was centrifuged at 1500 rpm for 10 min at 4 °C, and the supernatant was aliquoted and stored at −80 °C for future use. ELISA was conducted for the measurement of VEGF concentrations.

IncuCyte Angiogenesis 96-well PrimeKit (Essen Bioscience, Ann Arbor, MI, USA, catalog# 4452) and IncuCyte Angiogenesis PrimeKit VEGF/Suramin Supplement Kit (Essen Bioscience, catalog# 4437) were used as per the manufacturer’s protocols with some modifications. IncuCyte Angiogenesis 96-well PrimeKit included normal human dermal fibroblasts (NHDFs), human umbilical vein endothelial cells—CytoLight Green (GFP-HUVECs), seeding media and seeding media supplement, growth media and growth media supplement, assay media and assay media supplement, and a 96-well assay plate. Briefly, on Day 1, NHDFs were seeded into 96-well plates at a density of 2430 cells per well in complete seeding medium composed of seeding media and seeding media supplement. After NHDFs settled for 1 h at room temperature (RT), GFP-HUVECs were seeded into the wells at a density of 1000–1500 cells per well in complete seeding medium. GFP-HUVECs were then allowed to settle at room temperature for an additional hour. Subsequently, the 96-well plates were placed into the IncuCyte S3 (Sartorius, Göttingen, Germany). After 30 min of warming up, the IncuCyte monitored the vascular network formation every 6 h under green and phase channels at 10× magnification with a standard scan type. On Day 2, the complete seeding medium was aspirated and replaced with complete growth medium composed of growth media and growth media supplement. On Day 3, the complete growth medium was removed, and treatments prepared in the complete assay medium composed of assay media and assay media supplement were added into the 96-well plates. The treatments included the following groups: (1) a serial dilution of VEGF in CCM-VEGF (8 ng/mL, 4 ng/mL, 2 ng/mL, and 1 ng/mL), (2) their respective amounts of CCM-control based on the volumes of CCM-VEGF, (3) 4 ng/mL of commercial VEGF as a positive control, and (4) 100 µM of suramin in the presence of VEGF as a negative control. Suramin, a general tyrosine kinase inhibitor, was used to block the VEGF pathway. Refeeding was conducted with fresh complete assay medium containing treatments every 2–3 days until Day 10 or Day 14. Image analysis was performed using the IncuCyte automated angiogenesis algorithm (Incucyte software, v2024B, Sartorius).

### 2.6. Isolation of Extracellular Vesicles

Mesenchymal stem cells (MSCs) in T150 flasks were transfected with *EGFP* mRNA or *VEGF* mRNA as described above. Four hours post-transfection, MSC growth medium containing the transfection reagent was removed, and cells were washed 3 times with DPBS (Gibco, catalog# 14190-250) to eliminate residual exosomes. Subsequently, 20 mL of EV harvesting medium composed of MEMα supplemented with 16.5% exosome depleted fetal bovine serum (Gibco, catalog# A2720801), 1% GlutaMAX and 1% Penicillin–Streptomycin solution was added into each flask. Control MSCs and transfected MSCs were incubated for an additional 48 h, after which the conditioned media were collected and centrifuged at 3000× *g* for 15 min to remove cell debris. The supernatant was transferred to a new conical tube, and ExoQuick-TC (System Biosciences, Palo Alto, CA, USA, catalog# EXOTC50A-1) was added at a 1:5 ratio. The mixture was refrigerated overnight at 4 °C, followed by centrifugation at 1500× *g* for 30 min at room temperature. After aspirating the supernatant, the extracellular vesicle pellet was resuspended in 25 mM trehalose prepared in 0.9% sodium chloride. Each EV pellet obtained from 1 million MSCs was resuspended in 100 µL of 25 mM trehalose. The EV suspension was aliquoted and stored at −80 °C for future use.

### 2.7. Nanoparticle Tracking Analysis Using NanoSight

Extracellular vesicle (EV) size and concentration were evaluated using the NanoSight NS300 system (Malvern Panalytical, Malvern, UK). Briefly, after mounting the laser module with the low-volume flow cell, the system was primed with Milli-Q water. To confirm proper system performance, 100 nm Latex Transfer Standard beads (Malvern Panalytical, catalog# NTA4088) were run prior to sample analysis. EV samples were diluted to 1:200 in DPBS and loaded into the system using 1 mL syringes connected to the NanoSight syringe pump (Malvern Panalytical, UK). The NanoSight NTA software (NTA3.4) was used to set up the sample loading rate, optimize camera settings, and analyze particle data. Results were exported as PDF files for documentation.

### 2.8. EV Surface Marker Detection by Flow Cytometry

Extracellular vesicle (EV) surface markers were analyzed using the MACSPlex Exosome Kit (human) (Miltenyi Biotec, Bergisch Gladbach, Germany, catalog#130-108-813) following the manufacturer’s protocols. EV samples derived from VEGF-MSCs and control MSCs were thawed on ice and diluted in MACSPlex Buffer according to their concentrations determined by nanoparticle tracking analysis (NTA). The samples mixed with MACSPlex Exosome Capture Beads were incubated overnight at room temperature, protected from light, on an Incu-Shaker Mini shaking incubator (Benchmark Scientific, Sayreville, NJ, USA) at 450 rpm. After incubation, the supernatant was removed by centrifugation at 3000× *g* for 5 min. MACSPlex Exosome Detection Reagents were added, and the samples were incubated for 1 h under the same conditions on an Incu-Shaker Mini shaking incubator. Following incubation, samples were washed once with MACSPlex Buffer, resuspended in 150 uL of MACSPlex Buffer, and then transferred to flow tubes. Data acquisition was performed with an uptake volume of 70 µL for each sample using the MACSQuant16 Flow Cytometer, and analysis was conducted using MACSQuant analysis software (Version 2.13.0). A MACSPlex Buffer only control was processed identically without the addition of EVs.

### 2.9. Negative Staining Transmission Electron Microscopy (TEM)

Isolated extracellular vesicles (EVs) were fixed at room temperature in freshly prepared 2% paraformaldehyde (Thermo Fisher Scientific, catalog# 043368.9M) for 20 min. Fixed EVs were applied to glow-discharged, formvar/carbon-coated 200-mesh copper grids (Electron Microscopy Sciences, Hatfield, PA, USA) and allowed to adhere for 5 min. The grids were briefly washed atop a drop of distilled H_2_O, followed by negatively staining atop a drop of 2% aqueous uranyl acetate. After air drying, grids were examined in TEM using a JEOL 1400 transmission electron microscope (JEOL USA, Peabody, MA, USA) operated at 80 kV and equipped with an AMT NanoSprint 12 camera (AMT, Woburn, MA, USA).

### 2.10. Extracellular Vesicle Internalization

Apoptosis of HUVECs was induced using staurosporine (STS) (Abcam, Waltham, MA, USA, catalog# AB120056). HUVECs were seeded into a 24-well glass-bottom plate (Cellvis, catalog# P24-1.5H-N) at a seeding density of 50,000 cells per well. After overnight incubation, apoptosis was induced by treating cells with 25 nM STS for 24 h, followed by washing twice with HUVEC growth medium lacking FBS to remove dead cells. EVs were added to the wells after labeling with an ExoGlow-Protein EV Labeling Kit (Red) (System Biosciences, catalog# EXOGP100A-1) according to the manufacturer’s protocol. Imaging was performed every 2 h for 14 h using Tomocube HT-X1 (Tomocube, Daejeon, Republic of Korea), and images were analyzed by TA viewer software (Tomocube, Republic of Korea, version 2.1.5) to assess EV internalization.

### 2.11. HUVEC Apoptosis Assay

HUVECs were seeded into a 96-well plate at a density of 7500 cells per well and incubated overnight to allow cell attachment. The following day, cells were treated with 25 nM STS in HUVEC growth medium for 24 h. To visualize apoptotic cells, IncuCyte Nuclight Rapid Red Dye (Sartorius, catalog# 4717) and IncuCyte Caspase-3/7 Green Dye (Sartorius, catalog# 4440) were added into the wells at a 1:1000 dilution. The red dye stained all HUVECs, while the green dye selectively labeled apoptotic cells containing activated caspase-3/7. The 96-well plate was placed in the IncuCyte S3 live cell imaging system and scanned every 1 to 2 h using 10× magnification under phase, green, and red fluorescence channels. After 24 h treatment, the medium containing STS and dyes was removed, and cells were washed twice with HUVEC growth medium lacking FBS. A serial dilution of EVs (50%, 20%, 10%, and 5%, *v*/*v*) or the corresponding amount of 25 mM trehalose was prepared in fresh HUVEC growth medium supplemented with exosome-depleted FBS and IncuCyte Caspase-3/7 Green Dye and then added into the wells. Here, a 5% EV treatment was prepared by mixing 5 µL of EV suspension in 25 mM trehalose with 95 µL of HUVEC growth media with supplements per well. Cells were monitored for an additional 30 h under the same imaging settings. IncuCyte basic analyzer was used for performing image analysis. Apoptotic cells were identified by yellow fluorescence, resulting from the colocalization of red and green signals.

### 2.12. Statistical Analysis

Data are presented as mean (SD). Statistical analyses were performed using two-way ANOVA with Tukey’s multiple comparisons test for angiogenesis assay and one-way ANOVA with Sidak’s multiple comparisons test for apoptosis assay. All calculations and analyses were conducted using Microsoft Excel and GraphPad Prism (Version 10.0.0). A *p* value less than 0.05 (*) was considered statistically significant.

## 3. Results

### 3.1. Kinetics of VEGF mRNA and Secreted Protein Post-Transfection

**Figure 1:**
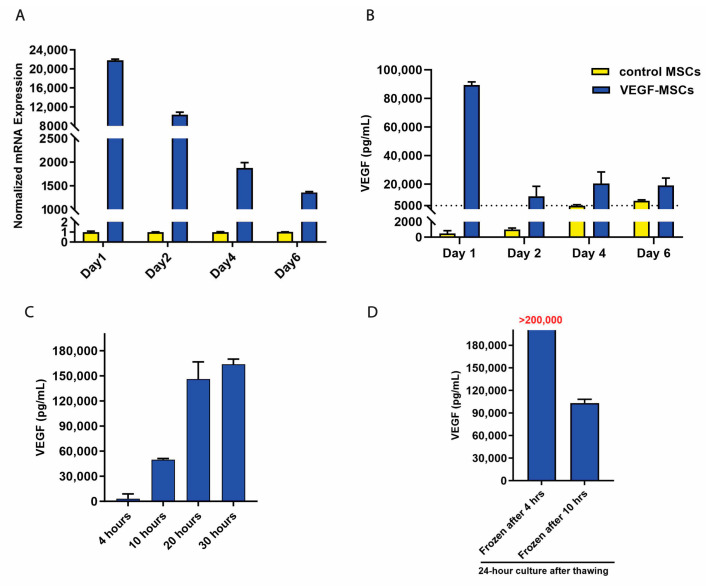
Kinetics of *VEGF* mRNA and protein levels in VEGF-MSCs. (**A**) *VEGF* mRNA levels detected by RT-qPCR. The values were normalized to *VEGF* mRNA levels of control MSCs at each time point. (**B**) Secreted VEGF levels in a 6-day time course experiment. One day post-transfection, conditioned culture media (CCM) were replaced with fresh MSC growth medium for all groups. No media change was performed from Day 2 to Day 6. (**C**) Secreted VEGF levels 4, 10, 20, and 30 h post-transfection. (**D**) VEGF protein levels 24 h after thawing VEGF-MSCs. After 4 or 10 h of transfection, VEGF-MSCs were cryopreserved, and VEGF levels in CCM were measured 24 h after thawing VEGF-MSCs. Data presented as mean (SD) (*n* = 3).

To enhance the therapeutic potential of extracellular vesicles derived from MSCs in ischemic conditions and diseases, we investigated the feasibility and efficacy of *VEGF* mRNA transfection into MSCs due to the angiogenic and anti-apoptotic properties of VEGF. In this study, we first conducted a time-course experiment over 6 days to evaluate VEGF expression dynamics. *VEGF* mRNA levels in transfected MSCs (VEGF-MSCs) and secreted VEGF protein levels in conditioned culture media (CCM) on Days 1, 2, 4, and 6 were detected by RT-qPCR and ELISA, respectively. Compared to control MSCs, *VEGF* mRNA levels in VEGF-MSCs were significantly elevated throughout the six days. Noticeably, *VEGF* mRNA level peaked on Day 1, reaching a level more than 20,000-fold higher than that of control MSCs (Figure 1A). Correspondingly, secreted VEGF protein levels in VEGF-MSCs (89.4 ng/mL) reached nearly 180-fold higher on Day 1 compared to control MSCs (0.5 ng/mL) (Figure 1B). After media replacement on Day 1, VEGF levels in CCM of VEGF-MSCs (11.5 ng/mL) remained elevated more than 10-fold on Day 2 compared to controls (1.0 ng/mL). However, VEGF protein levels were only a four-fold increase on Day 4 and a two-fold increase on Day 6 compared to control levels (Figure 1B). Although there was a more than 1000-fold increase in *VEGF* mRNA of VEGF-MSCs on Day 4 and Day 6, protein levels were not elevated correspondingly, indicating potential transfected mRNA degradation or translational inefficiency beyond 2 days post-transfection. The results confirm that *VEGF* mRNA can be successfully introduced into MSCs and demonstrate VEGF protein overexpression in VEGF-MSCs peaks within one day after transfection, with a low level of overexpression after 2 days. This transient expression profile suggests a potential time window of EV harvesting of 1–2 days post-transfection.

Next, to determine the optimal timing for cryopreserving cells for future use or to remove media containing transfection reagent to maximize the collection of CCM enriched with VEGF proteins, we further evaluated the VEGF protein expression pattern over a 30 h period. As shown in Figure 1C, secreted VEGF levels were low (3444 pg/mL) at 4 h post-transfection. However, a significant increase was observed at 10 h post-transfection, and the fastest rise occurred between 10 and 20 h. This suggests that VEGF protein expression in CCM of VEGF-MSCs peaks within 20 h. Furthermore, to assess whether 4 h incubation or 10 h incubation with transfection reagents is sufficient to retain high VEGF mRNA and protein levels, VEGF-MSCs were cryopreserved at both 4 and 10 h post-transfection, then thawed and cultured for an additional 24 h. Notably, VEGF protein levels for cells harvested at 4 h were remarkably higher than the cells harvested at 10 h (Figure 1D). These results indicate that the 4 h time point is optimal for either cryopreserving cells or removing media with transfection reagents to preserve robust VEGF secretion. In conclusion, these findings highlight that CCM containing EVs could ideally be collected between 4 h and 2 days post-transfection to ensure maximal VEGF content.

### 3.2. Characterization of VEGF-MSCs and Angiogenic Effects of VEGF Secreted from VEGF-MSCs

Despite EV treatment as a cell-free therapy, EVs preserve many properties from parent MSCs. Characterization of MSCs was conducted in this study. MSCs are generally characterized by their fibroblast-like shape, surface marker expression, and trilineage differentiation potential. To assess whether transfection of *VEGF* mRNA and overexpression of VEGF protein changed the properties of MSCs, we have investigated the following three characteristics of VEGF-MSCs: (1) osteogenic and adipogenic potential (Figure S1A), (2) surface molecule expression (positive markers, CD90, CD105, and CD73; negative markers, CD14, CD19, CD34, CD45, and HLA-DR) (Figure S1B), and (3) proliferation rate (Figure S1C). In addition, an in vitro tumorigenicity assay was conducted, and no colonies from the groups of control MSCs and VEGF-MSCs were observed in the soft agar (Figure S1D). These results indicate that VEGF-MSCs are able to differentiate into osteo- and adipo-lineage cells, maintain their surface marker features, and proliferate normally with no signs of associated malignant transformation.

**Figure 2:**
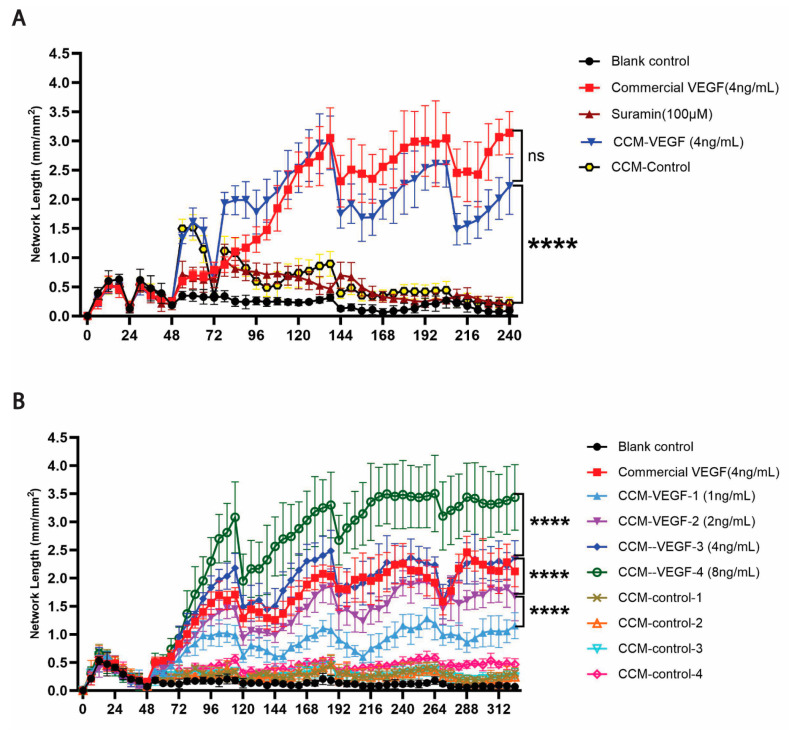
Angiogenesis induced by VEGF secreted from VEGF-MSCs. Human umbilical vein endothelial cells labelled by CytoLight Green (GFP-HUVECs) were used for the development of angiogenic networks, and the length of networks was determined by the IncuCyte live-cell analysis system. (**A**) VEGF effectively induced the formation of vascular networks, which was able to be inhibited by suramin, a VEGF signaling inhibitor. Data shown as mean (SD) (*n* = 6). (**B**) A concentration-dependent effect was shown in the angiogenesis assay using CCM-VEGF with different VEGF concentrations (1, 2, 4, and 8 ng/mL). Blank control, without any VEGF supplement added; suramin group, 4 ng/mL VEGF and 100 µM suramin added. Data shown as mean (SD) (*n* = 4); ns, not significant; **** *p* < 0.05.

Afterward, we investigated the biological activity of VEGF secreted from VEGF-MSCs using an angiogenesis assay. The effects of VEGF on angiogenesis were evaluated by the measurement of vascular network length. In this assay, suramin, a VEGF signaling inhibitor, effectively suppressed network formation (Figure 2A). VEGF (4 ng/mL) in CCM derived from VEGF-MSCs (CCM-VEGF) had comparable angiogenic effects with 4 ng/mL of commercial VEGF. Furthermore, CCM-VEGF (4 ng/mL VEGF) significantly enhanced the length of endothelial cell networks compared to CCM derived from control MSCs (CCM-control) (Figure 2A). Furthermore, CCM-VEGF preparations with different concentrations of VEGF (1, 2, 4, and 8 ng/mL) were tested (Figure 2B and Video S1). Predictably, each CCM-VEGF group produced significantly longer networks than its respective control. CCM-VEGF, containing the highest concentration of VEGF (8 ng/mL), promoted the fastest formation of vascular networks, achieving the longest networks at the end of the experiment. Additionally, the formation of angiogenic networks was noticeably concentration-dependent (CCM-VEGF-1 vs. CCM-VEGF-2, *p* < 0.0001; CCM-VEGF-2 vs. CCM-VEGF-3, *p* < 0.0001; CCM-VEGF-3 vs. CCM-VEGF-4, *p* < 0.0001). Collectively, the results indicate that VEGF released by VEGF-MSCs is biologically functional.

### 3.3. Characterization of EVs

Following confirmation of optimal VEGF secretion timing and VEGF protein functionality, EVs derived from MSCs were isolated and characterized. MSC growth medium containing transfection reagents was removed 4 h after transfection. control MSCs and VEGF-MSCs were cultured in EV harvesting medium for 48 h, after which CCM was collected for EV isolation. EVs were isolated using ExoQuick-TC and resuspended in 25 mM trehalose, a cryoprotectant commonly used to enhance EV stability and prevent EV aggregation [22].

**Figure 3:**
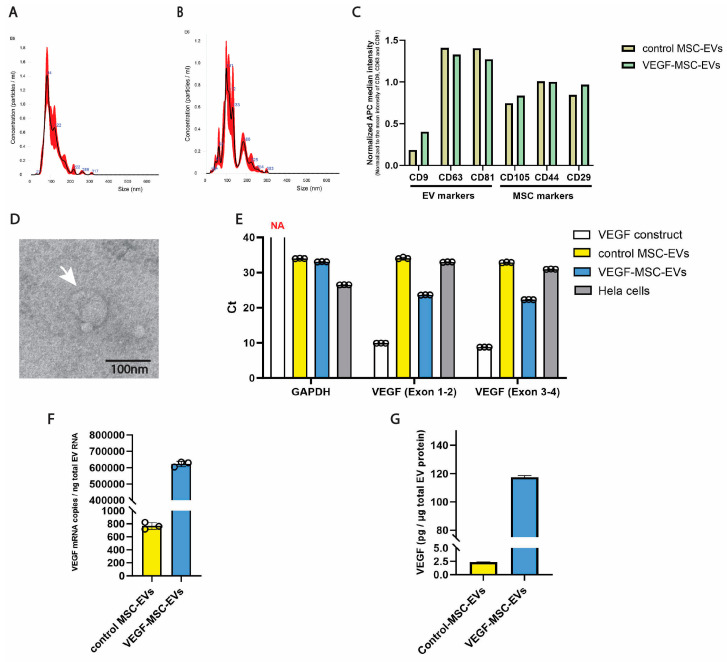
Characterization of extracellular vesicles. Control MSC-EVs (**A**) and VEGF-MSC-EVs (**B**) were assessed by nanoparticle tracking analysis (NTA), revealing a peak diameter of approximately 100 nm. Red shading represents standard error between video captures. (**C**) EV surface epitopes were analyzed using the MACSPlex Exosome Kit by flow cytometry. Both control MSC-EVs and VEGF-MSC-EVs exhibited positive expression of typical EV-associated markers (CD9, CD63, and CD81) and MSC-associated markers (CD105, CD44, and CD29). (**D**) Transmission electron microscopy of isolated EVs. The white arrow identifies a typical MSC-derived EVs. Scale bar: 100 nm. (**E**) Ct values of oligo (dT)-primed RT-qPCR. The *VEGF* mRNA construct served as a negative control for the *GAPDH* primers and as a positive control for two *VEGF* primer sets, *VEGF* (Exon 1–2) and *VEGF* (Exon 3–4). Hela cell total RNA was used as a positive control for the *GAPDH* primers. VEGF-MSC-EV mRNA exhibited significantly lower Ct values with both of *VEGF* primer sets compared to control MSC-EV mRNA. No Ct value for *GAPDH* was detected in the *VEGF* mRNA construct sample (NA, not available). Data are presented as mean (SD) (*n* = 3). (**F**) *VEGF* mRNA copy number with *VEGF* (Exon 3–4) primers. Data are presented as mean (SD) (*n* = 3). (**G**) ELISA quantification of VEGF protein levels. Data are presented as mean (SD) (*n* = 2).

The results of nanoparticle tracking analysis (NTA) revealed an average (±standard error, SE) concentration of 1.60 × 10^10^ (±1.38 × 10^9^) particles/mL for EVs derived from control MSCs (control MSC-EVs) with an average (±SE) diameter of 110.1 (±0.9) nm, and 1.16 × 10^10^ (±7.7 × 10^8^) particles/mL for EVs derived from VEGF-MSCs (VEGF-MSC-EVs) with an average size 128.6 (±2.9) nm (Figure 3A,B). For surface marker analysis, 1 × 10^9^ particles from each sample were used. Both control MSC-EVs and VEGF-MSC-EVs expressed typical EV markers (CD9, CD63, and CD81) and MSC surface markers (CD105, CD44, and CD29) (Figure 3C). Transmission electron microscopy (TEM) demonstrated that isolated EVs showed a typical round cup-shaped morphology (Figure 3D). The presence of VEGF mRNA and protein in EVs was next confirmed by RT-qPCR and ELISA analyses. To assess the integrity of *VEGF* mRNA loaded to EVs, oligo (dT)-primed RT-qPCR was performed using two sets of *VEGF* primers spanning *VEGF* mRNA exon 1–2 and exon 3–4 junctions. Comparable Ct values were observed for primers *VEGF* (Exon 1–2) and primer *VEGF* (Exon 3–4) (average Ct: 23.64 vs. 22.31), indicating preserved mRNA integrity. Notably, compared to control MSC-EVs, VEGF-MSC-EVs exhibited significantly lower Ct values with both primer sets (*VEGF* (Exon 1–2): average Ct 23.64 vs. Ct 32.99; *VEGF* (Exon 3–4): average Ct 22.31 vs. Ct 30.95), demonstrating a significantly increased abundance of *VEGF* mRNA in VEGF-MSC-EVs (Figure 3E). Furthermore, the *VEGF* mRNA copy number was calculated using a standard curve generated from the *VEGF* mRNA construct with *VEGF* (Exon 3–4) primers (Figure S2). As shown in Figure 3F, the *VEGF* mRNA copy number in VEGF-MSC-EVs was approximately 800-fold higher than that in control MSC EVs. Additionally, ELISA analysis showed that VEGF protein levels in VEGF-MSC-EVs were approximately 50-fold higher than control MSC-EVs after normalization to total EV protein content (Figure 3G). Collectively, VEGF-MSC-EVs contained VEGF mRNA and protein and retained the characteristic EV size distribution and surface marker profile.

### 3.4. Superior Anti-Apoptotic Effects of VEGF-MSC-EVs in HUVECs

Under ischemic conditions, preserving the survival of endothelial cells is critical to maintain adequate blood flow and prevent tissue damage. To assess the anti-apoptotic potential of EVs, we developed a human umbilical vein endothelial cell (HUVEC) apoptosis assay. HUVECs, a well-characterized cellular model for investigating venous endothelial function, were treated with 25 nM staurosporine (STS) to induce apoptosis for 24 h. Following STS removal, the number of apoptotic cells in the wells was confirmed to be very low with no statistically significant differences between wells (Figure S3), and then EVs were added to evaluate their capability to delay HUVEC apoptosis.

**Figure 4:**
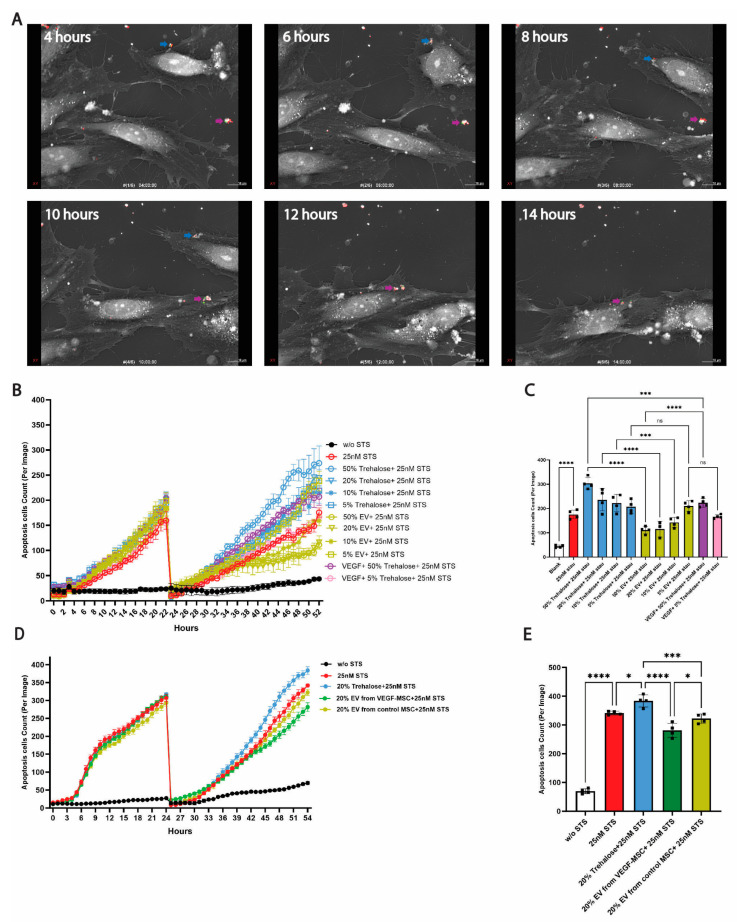
VEGF-MSC-EVs exhibit superior anti-apoptotic effects on HUVECs. (**A**) Internalization of EGFP-MSC-EVs by HUVECs. EGFP-MSCs derived EVs (EGFP-MSC-EVs) labeled with ExoGlow-Protein (Red) were incubated with HUVECs pretreated with 25 nM staurosporine (STS). Real-time 3D imaging was performed using the Tomocube HT-X1 for 14 h. Blue and purple arrows indicate two distinct EV clusters. Scale bar: 10 µm. Apoptosis in HUVECs was induced by 25 nM staurosporine (STS), and apoptotic cells were visualized by colocalization of Nuclight Rapid Red Dye and Caspase-3/7 Green Dye. (**B**) MSC-EVs protected HUVECs from apoptosis in a dose-dependent manner. (**C**) Quantification of apoptotic cells 30 h post-EV treatment. The protective effects of EVs were observed at 50%, 20%, and 10% EV concentrations, while 5% EVs and equivalent volumes of vehicle controls (50%, 20%, 10%, and 5% 25 mM Trehalose) did not promote HUVEC survival. Blank control, without adding STS. Positive control, with 100 ng/mL commercial VEGF added. (**D**) VEGF-MSC-EVs demonstrated superior anti-apoptotic effects compared to EVs derived from control MSCs. (**E**) Quantification of apoptotic cells 30 h post-treatment with VEGF-MSC-EVs. An ordinary one-way ANOVA followed by Sidak’s multiple comparisons test was used for statistical analysis. Data shown as mean (SD) (*n* = 4); ns, not significant; * *p* < 0.05; *** *p* < 0.001; **** *p* < 0.0001.

To confirm MSC-EVs uptake by HUVECs, EGFP-MSC-derived EVs (EGFP-MSC-EVs) labeled with ExoGlow-Protein (Red) were incubated with HUVECs pretreated with 25 nM STS. Real-time 3D imaging demonstrated progressive internalization of EV clusters with green and red fluorescence signals (Figure 4A). A 3D multiplanar reconstruction visualized the internalized EVs in an HUVEC at 14 h post-incubation (Video S2).

An initial HUVEC apoptosis experiment confirmed that control MSC-EVs exert dose-dependent anti-apoptotic effects. HUVEC growth medium supplemented with exosome-depleted FBS containing 50%, 20%, 10%, and 5% (*v*/*v*) control MSC-EVs (corresponding to 8 × 10^9^, 3.2 × 10^9^, 1.6 × 10^9^, and 0.8 × 10^9^ particles/mL, respectively) was evaluated. A 5% EV treatment was prepared by mixing 5 µL of EV suspension in 25 mM trehalose with 95 µL of HUVEC growth media with supplements per well, corresponding to the amount of EVs secreted by 50,000 MSCs. As shown in Figure 4B,C, the treatments with 50%, 20%, and 10% control MSC EVs significantly delayed HUVEC apoptosis 30 h post-treatment compared to the corresponding trehalose vehicle controls. In contrast, 5% EV treatment did not show the protective effects. Additionally, the 50% trehalose vehicle supplemented with 100 ng/mL VEGF improved HUVEC survival, but the effect was less pronounced than 50% EV treatment. Furthermore, the comparison between VEGF-MSC-EVs (20%, corresponding to 2.32 × 10^9^ particles/mL) and control MSC-EVs (20%, corresponding to 3.32 × 10^9^ particles/mL) revealed that both conferred the anti-apoptotic benefits. VEGF-MSC-EV (20%) treatment reduced apoptotic cells by 26.6% compared to vehicle control, and control MSC-EV (20%) treatment decreased by 16.0%. Notably, VEGF-MSC-EVs were more effective than controls, decreasing apoptotic cells by 12.6% compared to control MSC-EVs (Figure 4D,E). Our findings highlighted that EV-mediated protection for HUVECs is in a concentration-dependent manner and that VEGF-MSC-EVs pose superior anti-apoptosis properties compared to control-MSC-EVs, supporting their potential for therapeutic applications in ischemia-associated conditions and diseases.

## 4. Discussion

Over the past decade, MSC-derived EVs (MSC-EVs) have gained increasing attention as a therapeutic tool for ischemic diseases. MSC-EVs have shown great promise in the treatment of ischemia-associated conditions and diseases, including ischemic stroke, myocardial infarction, wound healing, renal ischemia reperfusion injury, ischemia-reperfusion injury of hearts, and other ischemia-associated conditions and diseases [4,23,24,25,26]. Despite MSC-EVs having inherited immunomodulatory, pro-angiogenic, and anti-apoptotic properties from parent MSCs, the clinical translation of MSC-EVs remains limited by challenges including low yield, inefficient therapeutic molecule loading, and limited targeting capabilities. To enhance the therapeutic efficacy of MSC-EVs in ischemic conditions, promoting angiogenesis and enhancing endothelial cell survival are essential strategies for alleviating ischemia-induced tissue damage. To our knowledge, our study is the first to generate VEGF-enriched EVs by engineering MSCs with *VEGF* mRNA. We comprehensively characterized both parent VEGF-MSCs and VEGF-MSC-EVs, confirming the elevated VEGF mRNA and protein levels in VEGF-MSC-EVs and their function in promoting HUVEC survival. This approach offers a novel avenue to enhance therapeutic efficacy of MSC-EVs for treating ischemia-associated conditions and diseases.

Engineering EV-producing MSCs has been investigated in preclinical studies for years as a promising cell-therapy approach. Given that VEGF is a pivotal angiogenic molecule, the combination of MSC therapy and *VEGF* mRNA transfection has been studied in the treatment of various diseases such as acute myocardial infarction [17], cardiac disease [27], critical limb ischemia [18], and fat graft transplant [28]. Despite variations in *VEGF* mRNA sources and transfection methods, all studies consistently reported that overexpressed VEGF protein expression peaked within 24 h after transfection, and remained at elevated levels for at least 4 days, which aligns with our results (Figure 1). Notably, only Yu et al. measured mRNA levels at 24 h time point [28], reporting a 70,000-fold increase compared to our 20,000-fold increase. Nevertheless, comparable VEGF protein concentrations (100–150 ng/mL) were observed at 24 h across studies. The discrepancy between VEGF mRNA and protein levels may be caused either by the limited essential materials required for protein synthesis or by distinct mRNA degradation rates under varying microenvironments [29]. Additionally, our study reported the detailed dynamic expression pattern of VEGF and characterized the innate features of VEGF-MSCs, including surface markers, differentiation potential, and proliferation rate. Importantly, we identified 4 h post-transfection as the optimal time point to switch to EV-harvesting medium, providing a foundation for standardizing EV production from VEGF-MSCs for future research and clinical applications.

Since MSC therapy primarily exerts its therapeutic benefits via paracrine effects, and EVs play a crucial role in the process, EV-based therapy is emerging due to advantages such as low immunogenicity, the capacity to cross biological barriers, and low tumorigenic risk [30]. MSC-derived EVs stand out because they inherit the beneficial features of their parent cells. To enhance the therapeutic potential of MSC-EVs as natural therapeutic carriers, a variety of approaches have been developed, including EV surface modification and loading of therapeutic molecules (nucleic acids and proteins). Different time points in the EV generation cycle can be leveraged to engineer EVs, including pre-EV isolation, post-EV isolation, and in vivo engineering EV generation [31]. Both pre-isolation and post-isolation approaches are widely used for genetic engineering of EVs [32,33]. Recent studies have reported *VEGF* mRNA delivery either by engineering parent cells or by directly loading synthetic transcripts into isolated EVs [34,35,36]. Pre-isolation engineering, as employed in this study, enables EVs to be naturally loaded through endogenous biogenesis pathways, which may better preserve EV membrane integrity and native structure. In contrast, post-isolation methods such as co-incubation with transfection reagents or sonication could potentially compromise EV membrane integrity. Moreover, engineering parent cells before EV isolation may result in variable cargo loading due to EV biogenesis, leading to high levels of certain target molecules but low or inconsistent levels of others, whereas post-isolation methods offer greater control over cargo quantity and composition.

In this study, we utilized an endogenous loading (pre-EV isolation) method by transfecting *VEGF* mRNA into EV-producing MSCs, thereby increasing VEGF mRNA and protein content in EVs to promote their angiogenic and anti-apoptotic properties. Previous work demonstrated that VEGF transfer from MSC-EVs to recipient cells is critical for the therapeutic effects of EVs [19]. A recent study has shown that fibroblast-derived EVs delivered *VEGF* mRNA effectively and rescued ischemic injury successfully. They transfected VEGF cDNA plasmids into EV-producing cells, resulting in high *VEGF-A* mRNA levels detected in EVs and robust VEGF-A protein expression in recipient cells [36]. We directly introduced *VEGF* mRNA into EV-producing MSCs in this study and demonstrated high levels of both VEGF mRNA and protein in the VEGF-MSC-EVs (Figure 3). Compared to cDNA plasmid transfection, the mRNA approach eliminates the risk of genetic rearrangement or insertional mutagenesis in parent MSCs, addressing safety concerns caused by introducing plasmid gene fragments in clinical applications. While our approach enables efficient loading of both mRNA and protein to engineered EVs, several translational challenges remain. First, the use of lipofectamine raises potential safety and regulatory concerns, and future efforts should focus on clinically compatible loading strategies such as electroporation. In addition, stringent quality control of synthesized mRNA is essential, and GMP-grade mRNA constructs should be employed. Furthermore, the long-term stability and transport of EV products remain practical hurdles as EV-based therapies require ultra-low temperature storage [37], and lyophilization may offer a promising solution. Together, these considerations could be critical for scalable and clinically viable engineered EV therapeutics.

VEGF, our target agent, was overexpressed in MSCs and their EVs by transfecting *VEGF* mRNA. To assess the bioactivity of overexpressed VEGF, we performed an in vitro angiogenic assay, which demonstrated that VEGF secreted by VEGF-MSCs exhibited proangiogenic effects comparable to commercial VEGF at equivalent concentration (Figure 2). Additionally, an HUVEC apoptotic assay we developed confirmed that VEGF-MSC-EVs effectively rescued HUVECs from apoptosis and were more potent than control MSC-EVs. Interestingly, treatment with 100 ng/mL commercial VEGF in 50% trehalose vehicle also improved HUVEC survival, but the effect was significantly less pronounced compared to the corresponding treatment of VEGF-MSC-EVs (Figure 4). This indicates that, beyond VEGF encapsulated in VEGF-MSC-EVs, other bioactive factors contribute to enhancing HUVEC survival, such as VEGF mRNA, long non-coding RNA (LncRNA), microRNA, or other proteins. For example, miR-100-5p from MSC-EVs has been shown to ameliorate ischemia reperfusion injury [25], and another study reported that MSC-EVs mitigate HUVEC senescence and promote angiogenesis via miR-146a rather than VEGF [38]. It remains unclear whether both *VEGF* mRNA and protein exert the anti-apoptotic effects in our study, and the underlying mechanism merits rigorous investigation. Additionally, MSC-EVs have inherent anti-inflammatory features mediated by miRNA, IL-10, or other immunomodulatory cargos [39,40]. These potential benefits of MSC-EVs highlight the advantages of natural EVs over synthetic nanoparticles, demonstrating biocompatibility and multiple therapeutic effects simultaneously. Overall, our study characterized VEGF-MSC-EVs and demonstrated their in vitro functionality, supporting the feasibility of enhancing MSC-EVs’ therapeutic efficacy by introducing *VEGF* mRNA into parent MSCs.

Although our findings are encouraging and provide new insights into enhancing MSC-EV therapeutic effects for ischemic diseases, further work remains before VEGF-MSC-EVs can be translated into clinical applications. First, the localization of VEGF mRNA and protein within EVs has not yet been elucidated. Although several studies have reported that VEGF can be either heparin-bound on the EV surface or associated with Hsp90 and remain highly stable in cancer-derived EVs, it remains unclear whether VEGF mRNA and protein in MSC-derived EVs are located on the vesicle surface or within the EV lumen [41,42]. Second, the precise mechanisms, whether through direct mRNA translation in recipient cells or via paracrine signaling, require further investigation. Third, rigorous preclinical studies are essential to validate the therapeutic efficacy and safety of VEGF-MSC-EVs in relevant ischemic models. Finally, scalable and standardized EV production methods must be developed to support clinical translation while addressing donor variability for producing the engineered EVs.

## 5. Conclusions

Our study provides a proof-of-concept for engineering MSC-EVs by transfecting *VEGF* mRNA into parent MSCs. This approach offers a novel avenue to augment the therapeutic potential of MSC-EVs in ischemia-associated conditions and diseases.

## Data Availability

The original contributions presented in this study are included in the article/Supplementary Materials. Further inquiries can be directed to the corresponding author.

## Supplementary Materials

The following supporting information can be downloaded at: https://www.mdpi.com/article/10.3390/cells15080717/s1, Figure S1: Characterization of VEGF-MSCs; Figure S2: Standard curve for RT-qPCR quantification of *VEGF* mRNA; Figure S3: Quantification of apoptotic cell following 24 h STS treatment; Video S1: Representative time-lapse video of CCM-VEGF (8 ng/mL) group on angiogenesis; Video S2: Representative 3D multiplanar visualization of EGFP-MSC-EVs internalized by an HUVEC at 14 h post incubation; Supplemental Materials and Methods.
