# Supplementary material for: Extracellular Vesicles Derived from VEGF mRNA-Engineered Mesenchymal Stem Cells Promote Endothelial Cell Survival
Source: Cells. 2026 Apr 18;15(8):717. doi: 10.3390/cells15080717 (PMC13115070; doi:10.3390/cells15080717)
Supplement: Supplementary file 1 [file cells-15-00717-s001.zip › cells-4196301-supplementary.pdf]

Supplemental Materials

Supplemental Figures

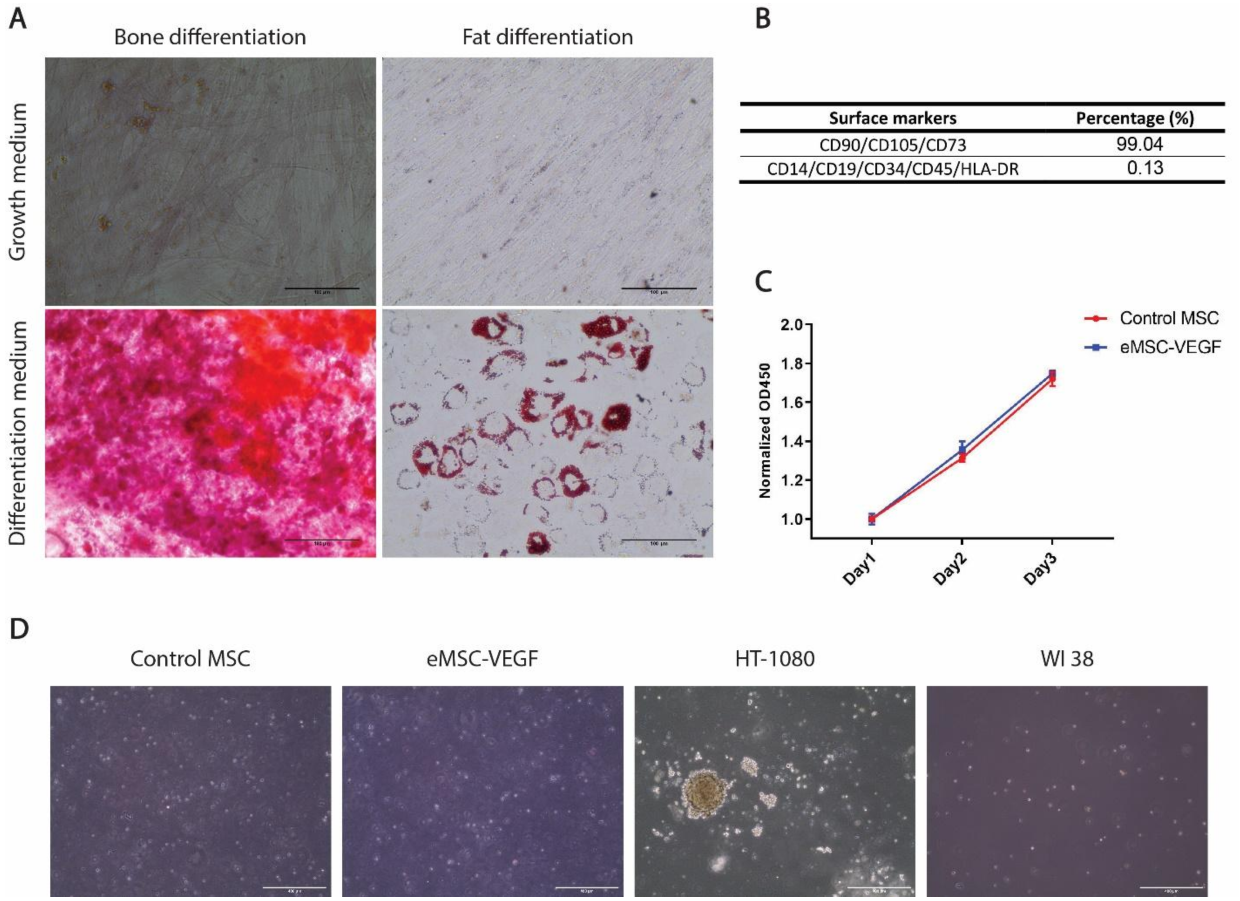

Figure S1 Characterization of VEGF-MSCs. A) The potential of VEGF-MSCs for bone and fat differentiation. Bone differentiation and fat differentiation were detected by alizarin red staining and oil red O staining, respectively. Scale bar:100 μm. **B)** Surface phenotypes of VEGF-MSCs. The presence of CD90, CD105 and CD73 and the absence of CD14, CD19, CD34, CD45 and HLA-DR were investigated by flow cytometry. **C)** Cell proliferation. The proliferation abilities of control MSCs and VEGF-MSCs were evaluated using CCK-8 assay. Data presented as mean ± SD (*n*=3). **D)** Tumorigenicity assay to rule out the oncogenic transformation of VEGF-MSCs. No colonies were observed in the cultures of control MSCs, VEGF-MSCs and WI 38 (a normal fibroblast cell line), while HT-1080 cells, a fibrosarcoma cell line, formed visible colonies after 14-day culture in soft agar. Scale bar: 400 μm.

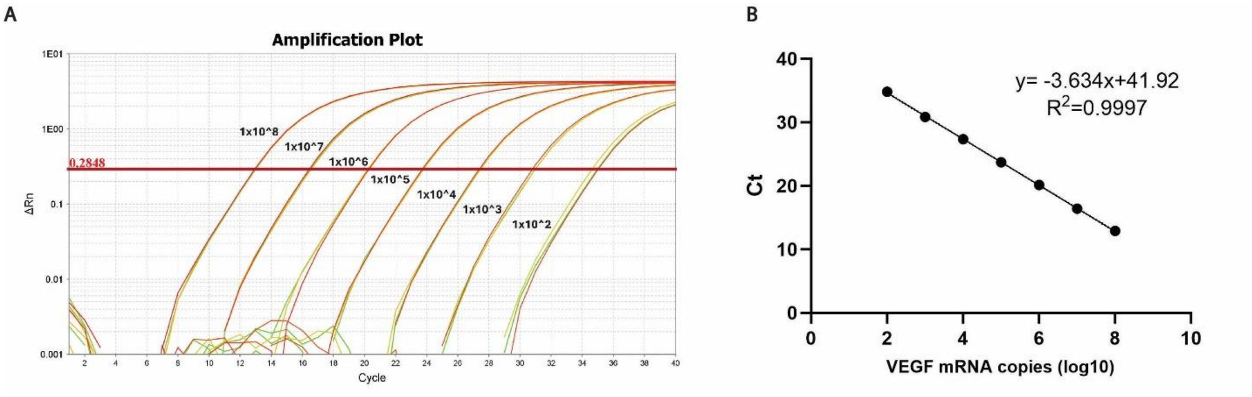

Figure S2 Standard curve for RT-qPCR quantification of VEGF mRNA. (A) Amplification plots for a 10-fold serial dilution of VEGF mRNA (TriLink), ranging from $1x10^8$ to $1x10^2$ copies per reaction. **(B)** Standard curve depicting the linear relationship between Ct values and the log_{10} of VEGF mRNA copy number: y=-3.634x+41.92, R2=0.9997. (*n*=3)

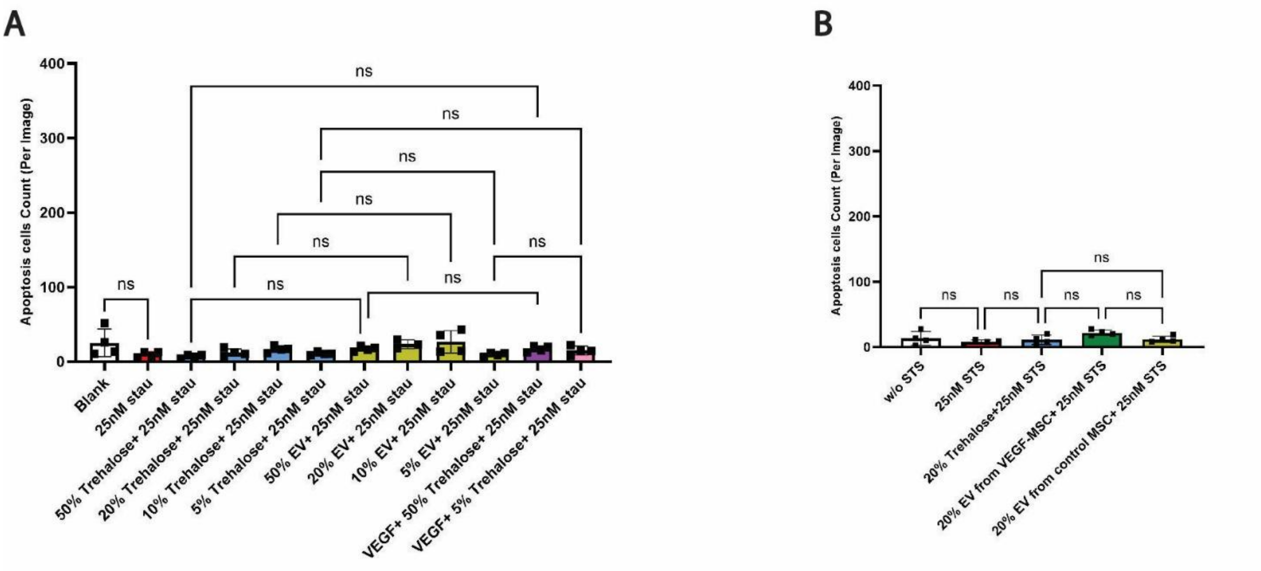

Figure S3 Quantification of apoptotic cell following 24-hour STS treatment. After 24 hours of STS exposure, the medium containing STS was removed, and cells were washed twice with FBS-free HUVEC growth medium. Apoptotic cell counts and statistical analyses were performed. **(A)** Apoptotic cell quantification prior to treatment with serial dilutions of EVs at concentrations of 50%, 20%, 10% and 5%. **(B)** Apoptotic cells quantification before administration of VEGF-MSC-EVs. One-way ANOVA followed by Sidak's multiple comparisons test was used for statistical analysis. Data shown as mean (SD) (*n*=4). ns, not significant.

Supplemental Videos

Video S1 Representative time-lapse video of CCM-VEGF (8 ng/mL) group on angiogenesis. The images were captured every 6 hours. Scale bar, 400 μm.

Video S2 Representative 3D multiplanar visualization of EGFP-MSC-EVs internalized by a HUVEC at 14 hours post incubation.

Supplemental Materials and Methods

Characterization of mesenchymal stem cells

The surface markers of MSCs were assessed using an MSC phenotyping kit (Miltenyi Biotec, catalog# 130-125-285), and the compensation of fluorescence spillover was conducted using MACS Comp Bead Kit (Miltenyi Biotec, catalog# 130-104-187) per the manufacturer's instructions. MACSQuant Analyzer 16 flow cytometer (Miltenyi Biotec, Bergisch Gladbach, Germany) was used for data collection, and data analysis was conducted using MACS Quantify software.

Osteogenic and adipogenic differentiation assays were performed using StemPro osteogenesis differentiation kit (Gibco, catalog# A1007201) and StemPro adipogenesis differentiation kit (Gibco, catalog# A10070-01), respectively. The differentiation media was changed twice a week. On day 14, oil red O solution (Sigma, catalog# O1391) was used for the visualization of lipid vacuoles in the adipogenic differentiation assay. On day 21, alizarin red staining solution (Sigma, catalog#TMS-008-C) was used to visualize the calcium deposits in the osteogenic differentiation assay.

Cell proliferation assay

The proliferation rate of mesenchymal stem cells was assessed using Cell Counting Kit-8 (CCK-8) (Dojindo, Catalog# CK04-13). On Day 0, cells were seeded into a 24-well plate at a density of 10,000 cells per well. The plate was incubated overnight to allow cells to attach. On Days 1, 2 and 3, a mixture of 40 μL of CCK-8 solution and 400 μL of MSC growth media was added into each well. The plate was incubated for 2 hours in the cell culture incubator, after which the absorbance at 450 nm was determined for triplicate wells each day using a microplate reader.

In-vitro tumorigenicity assay

In this assay, 67,000 cells were seeded per well in 6-well plates. VEGF-MSCs and control MSCs were cultured in 0.3% upper agar (SeaPlaque GTG Agarose, Lonza, Catalog # 50111) in MEMα supplemented with 5% human platelet lysate and 1x GlutaMAX. HT-1080 (ATCC, catalog# CCL-121) and WI38 (ATCC, catalog# CCL-75) were plated in 0.3% upper agar in DMEM supplemented with 10% FBS. HT-1080 is a human fibrosarcoma cell line used as a positive control, while WI 38 is a human normal fibroblast cell line used as a negative control for this soft agar colony formation assay. The bottom agar was composed of 0.5% SeaPlaque GTG

Agarose in the respective types of medium. The plates were placed into a cell culture incubator after the agar was solidified. To prevent desiccation of the upper agar, 100 μL of corresponding culture media were added into each well every 3-4 days. After a 14-day culture, images were captured to visualize colony formation.
